# Bone Regenerative Potential of Cross-Linked Collagen Membrane in Peri-Implant Osseous Defect: Case Series with Histologic/Micro-Computed Tomographic Findings

**DOI:** 10.3390/medicina59010176

**Published:** 2023-01-15

**Authors:** Won-Bae Park, Gazelle Jean Crasto, Ji-Young Han, Philip Kang, Hyun-Chang Lim

**Affiliations:** 1 Private Practice in Periodontics and Implant Dentistry, Seoul 02771, Republic of Korea; 2 Division of Periodontics, Section of Oral, Diagnostic and Rehabilitation Sciences, College of Dental Medicine, Columbia University, #PH7E-110, 630 W. 168 St., New York, NY 10032, USA; 3 Department of Periodontology, Division of Dentistry, College of Medicine, Hanyang University, Seoul 04763, Republic of Korea; 4 Department of Periodontology, Kyung Hee University College of Dentistry, Periodontal-Implant Clinical Research Institute, Kyung Hee University Medical Center, Seoul 02447, Republic of Korea

**Keywords:** bone regeneration, bone transplantation, dental implant, histology

## Abstract

The role of a barrier membrane is crucial in guided bone regeneration (GBR) for space creation and cell occlusiveness. Those properties of the membrane should be sustained for a sufficient period. For such purpose, several cross-linked collagen membranes were introduced and demonstrated favorable clinical outcomes. However, histologic data were not sufficient to support the effect of cross-linked collagen membranes. In the present case series, healing after GBR using a cross-linked collagen membrane was investigated in-depth via histologic and micro-computed tomographic (micro-CT) analyses. 1-ethyl-3-(3-dimethylaminopropyl) carbodiimide cross-linked collagen membrane was used in GBR for treating various peri-implant bone defects in seven patients. After 4–7 months of healing, newly formed tissue of hard consistency was observed over the implant platform. This tissue was carefully harvested and assessed. In micro-CT and histological analyses, evident new bone formation was revealed, especially in the vicinity of the collagen membrane. Moreover, it was histologically found that some newly formed bone was in intimate contact with the membrane. Although the exact mechanism of bone regeneration in the present cases was not clearly elucidated, the cross-linked collagen membrane appeared to contribute to ossification in GBR. Further studies are needed to confirm the findings of the present case series.

## 1. Introduction

Guided bone regeneration (GBR), described by Dahlin et al., 1998, involves the process of bone regeneration principally based on the understanding that different migratory patterns occurs for different cellular components in tissue healing [1]. Under such a notion, a mechanical barrier, such as barrier membranes, is applied to make the bone defect site separate from the adjacent tissue, aiming to exclusive migration of bone-forming cells to the bone defect (without soft tissue migration/penetration to the defect) [2].

In classic GBR, a non-resorbable expanded-polytetrafluoroethylene (e-PTFE) membrane was mainly used. Due to high cell-occlusive properties, such membranes demonstrated bone regeneration even without the support of bone substitute material [3]. Moreover, the addition of a titanium frame to the e-PTFE membrane allowed for more significant space-maintaining capability [4]. An extensive amount of literature exhibited the excellent regenerative potential of the e-PTFE membrane [5], leading to the e-PTFE membrane as a gold standard.

However, clinicians gradually sought resorbable membranes due to technical difficulty and the high complication rate of the e-PTFE membrane [6]. Consequently, collagen membranes are nowadays chosen in most GBR procedures [7]. In line with such a trend, clinical studies regarding collagen membranes demonstrated favorable clinical outcomes similar to the e-PTFE membrane [8,9,10]. Moreover, the complication rate of collagen membranes is markedly reduced compared to the e-PTFE membrane [11]. Nonetheless, the biodegradation of collagen membrane in the body can be a significant shortcoming [12] because that permits unwanted cells into the bone defect. To overcome such weakness, cross-linking technology was developed to modify the biomechanical characteristics of the collagen fibers [13]. Degradation of cross-linked collagen membranes becomes prolonged, and enough time for sufficient bone regeneration can be obtained [14,15]. A recent systematic review exhibited that cross-linked collagen membranes led to more vertical bone fill than non-cross-linked ones [16].

To ensure the effect of cross-linked collagen membranes, histologic evidence should be supplemented, especially from human histological samples. However, limited data have been provided due to the study design, ethical reasons, and clinical situations. Herein, we could obtain histologic samples non-invasively to determine the effect of a cross-linked collagen membrane. The aim of the present case series was to investigate the bone regenerative potential of the cross-linked collagen membrane histologically and with micro-computed tomography (micro-CT).

## 2. Case Presentation

In this case series, 7 patients requiring extraction of a periodontally compromised tooth (teeth), and dental implant treatment were included. All patients underwent periodontal treatment before implant treatment. Demographic information is presented in Table 1.

### 2.1. Surgical Procedure

#### 2.1.1. Implant Placement and GBR

**Figure 1:**
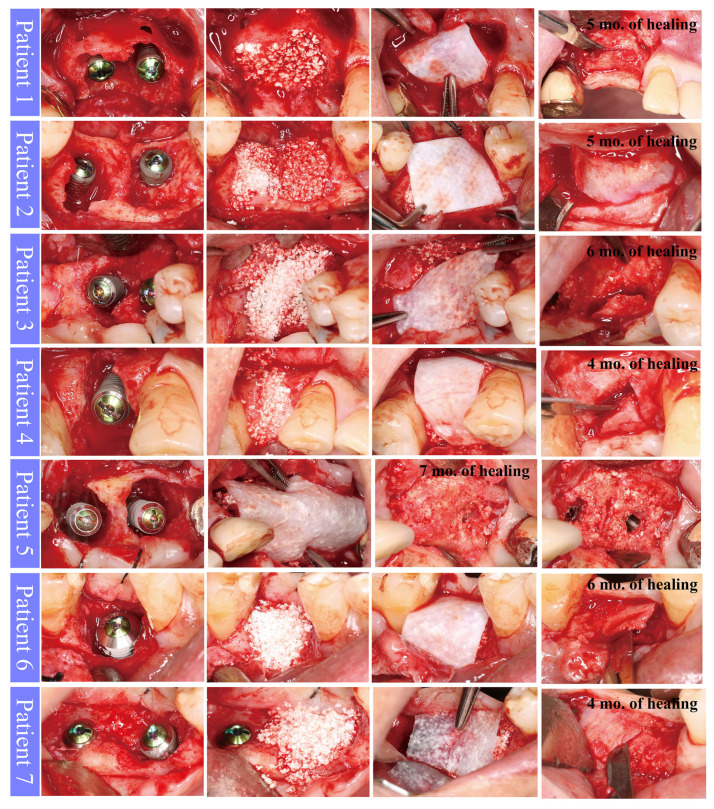
Representative clinical photographs of each case.

Implant placement timing was empirically determined, depending on the gingival tissue condition, the dimension of the bone defect/extraction socket, the state of inflammation and whether the primary stability of implants could be obtained in a proper position (3 patients: immediate implant placement, 4 patients: 2 months after tooth extraction). Surgical procedures applied to the patients were as follows. Under local anesthesia with 2% lidocaine containing 1:100,000 epinephrine (Huons, Seongnam, Republic of Korea), tooth extraction and thorough granulation tissue removal were performed. An osteotomy was carefully performed according to the manufacturer’s guidelines (Dentium, Suwon, Republic of Korea). Due to pre-operative alveolar bone destruction, the peri-implant bone defect was found after implant placement (Implantium, Denitum). Then, synthetic bone substitute material (biphasic calcium phosphate; Osteon III, Genoss, Suwon, Republic of Korea) was grafted to the defect. Slight over-augmentation was performed to cover the implant platform (approximately 2 mm from the platform). The augmented area was covered with a cross-linked (using 1-ethyl-3-[3-dimethylaminopropyl] carbodiimide; EDC) collagen membrane (Genoss, Suwon, Korea) made of bovine type I collagen. The membrane was stabilized by tucking it into the flap (Figure 1). Periosteal releasing incision was performed for tension-free flap closure. The flaps were sutured using Nylon material. Antibiotics (Cefradine 500 mg, Yuhan Pharmaceutical Co., Seoul, Republic of Korea) and anti-inflammatory drugs (Etodol^®^ 200 mg, Yuhan Pharmaceutical Co.) were prescribed for 7–10 days. The patient was recommended to gargle with 0.12% chlorhexidine solution (Hexamedine, Bukwang Pharmaceutical, Seoul, Republic of Korea) twice a day for two weeks. The suture material was removed after 7–10 days.

#### 2.1.2. Uncovering Procedure

After 4–7 months, healing abutments were connected to the implants. In all cases, bone-like tissue covered the implant platform. To access the cover screw, that tissue was removed using a #15 Bard-Parker blade. No exposure of the implant thread was found. A healing abutment was connected to the implant. The removed tissue was immersed in 10% buffered formalin for histological evaluation. The flaps were closed around the abutment. Antibiotics and anti-inflammatory drugs were administered for 5 days.

### 2.2. Micro-Computed Tomographic (Micro-CT) Examination and Histologic Evaluation

The specimen was fixed in a neutral buffered formalin solution (Sigma Aldrich, St. Louis, MO, USA). Then, a micro-CT (Skyscan 1173, Aartselaar, Belgium) scan was performed. After the specimen was decalcified, it was embedded in paraffin. The specimen was sectioned to a thickness of 5 μm, followed by H-E and Masson’s trichrome staining. The stained specimens were digitally scanned by a digital scanner (Panoramic 250 Flash III; 3 DHISTECH, Budapest, Hungary). The histologic observation was performed using computer software (CaseViewer ver 2.3; 3 DHISTECH).

## 3. Results

### 3.1. Clinical Findings

After 4–7 months of healing, the surgical sites were uncovered to connect healing abutments. No infection and wound dehiscence were observed in all patients during the healing period. A varying amount of the remnant of the collagen membrane was observed after flap reflection. Regenerated tissue was observed over the implant platforms in all patients. Peri-implant defects were also completely filled with the newly formed tissue (Figure 1). The regenerated tissue over the implant platform was hard in consistency, like bone tissue. This tissue was resistant to periodontal probing.

### 3.2. Micro-CT Findings

**Figure 2:**
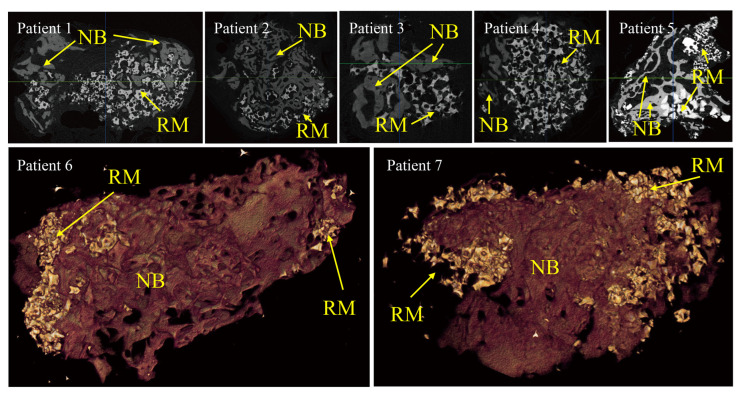
Micro-computed tomographic views of each case. Three-dimensional reconstructed images are provided for the samples from patients 6 and 7. NB indicates newly formed bone. RM indicates residual bone substitute material. In all specimens, the radio-opacity of residual bone substitute material is higher than that of newly formed bone.

In all specimens, a larger area of newly formed bone was found in the coronal part of the specimen (adjacent to the collagen membrane) compared to the apical part. The newly formed bone was well-mineralized. Bone substitute material and new bone tissue were clearly discerned due to differences in radio-opacity. The newly formed bone was well integrated with bone substitute material (Figure 2).

### 3.3. Histological Findings

Histologically, all specimens consisted of newly formed bone (lamellar bone, woven bone), bone substitute particles, and fibrovascular tissue. In five specimens (patients 1, 2, 3, 4, and 7), the remnant of the collagen membrane was observed. Generally, inflammatory cells were rarely observed. In patient 5, multinucleated giant cells were observed, but no notable inflammatory cell infiltration was found. 

At the coronal part of the specimen (especially near the location of the collagen membrane), lamellar bone developing thick trabecular was observed. Interestingly, some newly formed bone was intimately located near the membrane. That lamellar bone showed osteocytes in the lacuna, demarcation lines, and lining of fibroblast-like cells on the outer surface, representing active bone remodeling. One specimen (patient 3) exhibited greater corticalization compared to others. 

**Figure 3:**
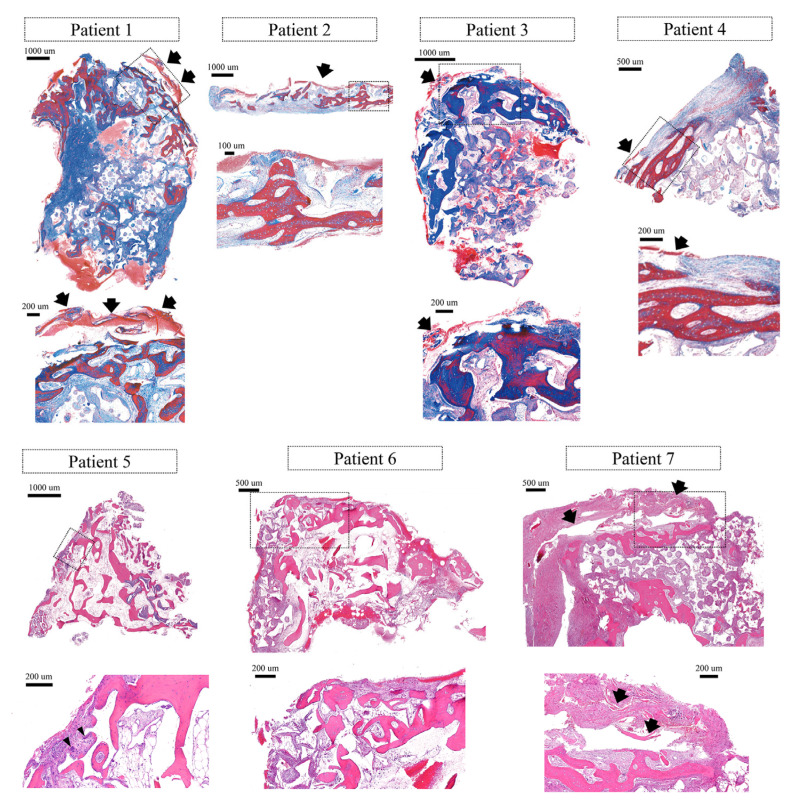
Histologic views of each case. Arrows indicate the remnants of a cross-linked collagen membrane. Arrowheads indicate multinucleated giant cells. Higher magnified views are presented for the box areas of the lower magnified views. Masson’s trichrome staining for the samples from patients 1–4. Hematoxylin-Eosin staining for the samples from patients 5–7.

At the apical part of the specimen, less mineralization was generally observed compared to the coronal part (near the collagen membrane). There, cell alignment, woven bone structure, and small lamellar bone fragments were seen on the bone substitute particles (Figure 3).

In the specimen from the longest healing (patient 5), bone marrow was observed (Figure 3).

The percentage of newly formed bone within the specimen ranged between 11.13% and 26.10%.

## 4. Discussion

In the present case series, GBR was performed using the cross-linked collagen membrane and synthetic bone substitute material in seven patients. After 4–7 months, newly formed tissue over the implant platform was examined, revealing hard tissue consistency clinically and remarkable new bone formation predominantly in the coronal area of the specimens (in the vicinity of the cross-linked collagen membrane) histologically and in micro-CT scan. Such findings were observed in all patients despite the varying size of the defect. 

The principles of GBR include space creation/maintenance, angiogenesis, wound stability, and primary wound closure [17]. Among them, a barrier membrane contributes to staging the space for bone regeneration [18]. Such space should be isolated long enough not to interfere with the bone-forming cascade. Despite a good amount of evidence supporting collagen membranes in GBR [7,19], large or complex defects may be challenging due to the biodegradation of the collagen membranes in the body [20]. However, the addition of cross-linking to the collagen membranes enhances bio-durability and mechanical properties [21], which may broaden the indication of collagen membrane application.

A variety of cross-linking techniques were developed. The collagen membrane in the present case series is cross-linked using EDC. In a rabbit calvarial defect model, the EDC-cross-linked collagen membrane presented favorable structural durability at 8 weeks of healing and greater new bone formation with the support of bone substitute material compared to the membrane alone and bone substitute alone [22]. In a dog model, that membrane contributed higher and long-lasting osteogenic activity, which was evaluated with an immunohistochemical assay [23].

In line with the above preclinical studies, the present case series demonstrated favorable bone regeneration in micro-computed tomographically and histologically. Especially the histologic outcome from human patients is valuable because most clinical studies regarding bone regeneration mainly demonstrated the radiographic appearance of bone formation. In this case series, new bone formation was distinct in the vicinity of the collagen membrane, which corroborates the clinical impression of hard consistency and high resistance when probing. Compared to the area adjacent to the membrane, the apical area presented less and still immature bone formation. Such indicates that the collagen membrane in the present case series contributed bone-forming cascade, behaving like an ossification assistant. Even though bone maturation is slow in the apical area from the membrane location, ossification near the membrane acts like an “exoskeleton.” It may provide an environment for ongoing bone formation.

The bone-forming characteristics in this case series were also reported in a ribose-cross-linked collagen membrane. In both clinical and preclinical studies, the ribose-cross-linked collagen membrane demonstrated ossification in proximity to or in close contact with the membrane [24,25]. Moreover, some parts of the membrane were invaded by osteoblastic cells [25]. From such results, the authors of the studies hypothesized “a reciprocal effect between the collagen matrix and osteoblasts or even undifferentiated cells that promote bone formation inside and outside” [25].

When using a non-resorbable membrane, the pseudoperiosteum layer is commonly found. The role of this layer has yet to be determined, but there were few cellular components in this layer histologically [26,27]. Such may be differentiated from the behavior of the cross-linked collagen membranes. It appears that the cross-linked collagen membrane performs not only space creation or protection of blood coagulum/bone substitute material but also positive biologic activity in the ossification process.

The clinical relevance of this case series is that clinicians do not have to aggressively remove the newly regenerated tissue to access the cover screw inserted into the implant. As this study demonstrated, that tissue may have newly formed bone (whether mature or immature), and maximum preservation of the tissue (for example, selective tissue removal for the abutment connection) would eventually contribute to the stability of marginal bone and overlying soft tissue. However, it should be taken in mind that the above may not be generalized in other cross-linked collagen membranes.

There were several limitations in the present study. Mainly, the limitations were derived from the nature of the study (case series), even though human histologic specimens relative to the cross-linked collagen membrane are hard to obtain. First, there was a small number of patients included. Second, the defect type, implant site, and implant placement timing were various and not standardized. Third, there was no control group, such as other collagen membranes. Fourth, histologic healing near the implant surface could not be revealed due to ethical reasons.

## 5. Conclusions

The present case series exhibited the potential of the cross-linked collagen membrane to contribute to ossification in GBR. However, one should keep in mind that there were no comparators in this case series. Therefore, further randomized clinical studies are needed to confirm the findings of the present case series (e.g., cross-linked vs non-cross-linked collagen membrane in large peri-implant defects involving histologic analysis).

## Figures and Tables

**Table 1:** Demographic information of the included patients.

Case	Age/Sex	Smoking	Systemic Diseases	GBR Sites	Implant Timing	Defect Morphology	Implant Diameter × Length	Wound Dehiscence/Membrane Exposure	Healing Period (Months)
1	73/M	No	No self-reported	#13 #14	immediate	wide gap defect, partial loss of septal bone, fenestration on the #13 area	3.8 × 12 3.8 × 12	No	5
2	59/M	No	Hypertension, Diabetes mellitus	#35 #36	immediate	wide gap defect, substantial loss of the buccal bone plate	4.3 × 10 4.8 × 10	No	5
3	72/F	No	No self-reported	#16 #17	immediate	exposure of thread on the coronal half of the #17 implant	4.3 × 10 4.3 × 10	No	6
4	75/M	No	No self-reported	#14	2 months after extraction	large dehiscence on the buccal and palatal aspects	4.3 × 12	No	4
5	67/M	Yes	No self-reported	#24 #25	2 months after extraction	wide gap defect, total loss of the buccal bone plate on the #24 implant	4.3 × 10 4.3 × 10	No	7
6	62/M	Yes	No self-reported	#46	2 months after extraction	gap defect with partial loss of the buccal bone plate	6.0 × 10	No	6
7	72/M	No	No self-reported	#46	2 months after extraction	gap defect on the coronal 1/3 of the #46 implant	4.3 × 10	No	4

## Data Availability

All data are included in the manuscript.
